# Neuroinflammatory Signaling and Immune Cell Infiltration Differ in Brains of Rats Exposed to Space Radiation and Social Isolation

**DOI:** 10.3390/life15050747

**Published:** 2025-05-06

**Authors:** Austin M. Adkins, Zachary N. M. Luyo, Alea F. Boden, Riley S. Heerbrandt, Richard A. Britten, Laurie L. Wellman, Larry D. Sanford

**Affiliations:** 1 Sleep Research Laboratory, Eastern Virginia Medical School, Macon & Joan Brock Virginia Health Sciences at Old Dominion University, Norfolk, VA 23501, USA; austin.adkins@cuanschutz.edu (A.M.A.); luyozn@odu.edu (Z.N.M.L.); bodenaf@odu.edu (A.F.B.); heerbrrs@odu.edu (R.S.H.); wellmall@odu.edu (L.L.W.); 2 Center for Integrative Neuroscience and Inflammatory Diseases, Eastern Virginia Medical School, Macon & Joan Brock Virginia Health Sciences at Old Dominion University, Norfolk, VA 23501, USA; brittera@odu.edu; 3 Biomedical and Translational Sciences, Eastern Virginia Medical School, Macon & Joan Brock Virginia Health Sciences at Old Dominion University, Norfolk, VA 23501, USA; 4 Radiation Oncology, Eastern Virginia Medical School, Macon & Joan Brock Virginia Health Sciences at Old Dominion University, Norfolk, VA 23501, USA

**Keywords:** social isolation, space radiation, immune cell infiltration, neuroinflammation, gene expression

## Abstract

Astronauts on the proposed Mars missions will be exposed to extended periods of social isolation (SI) and space radiation (SR). SI and SR-induced immune dysregulation can result in persistent neuroinflammation and neuronal damage which could negatively impact an astronaut’s health and ability to maintain adequate levels of performance. The synergistic effects of combined SI and SR on immune system functionality and the brain remain unknown. Determining how single and combined inflight stressors modulate the immune system is crucial for fully understanding pathways impacting astronaut health and performance. We used ground-based analogs of SI and SR in rodent models to investigate how SI and SR, and their combination (dual flight stressors (DFS)), impact immune cell recruitment into the brain and alter gene expression related to immune signaling and neuroinflammation. We also assessed whether putative phenotypic differences in stress resilience and vulnerability were reflected in neuroinflammatory-related gene expression. SI rats exhibited differences in neuroinflammatory signaling but no differences in infiltrating cells compared to Controls. SR rats exhibited up-regulated gene expression related to cytokine signaling and immune cell recruitment and unexpectedly depleted infiltrating immune cells. Many deficits related to the immune response in the SR animals were attenuated by dual exposure to SI. These data demonstrate significant differences in the effects of spaceflight stressors on immune function and how they may vary with individual stress resilience and vulnerability.

## 1. Introduction

Space exploration such as NASA’s proposed Mars missions will require crew members to serve on longer missions and travel deeper into space than ever before. Astronauts on these missions will experience prolonged exposure to multiple inflight stressors along with other challenges and obstacles that can pose significant risk to astronaut health and performance [1]. Interactions between stress and immune systems are well-established. In addition to hypothalamic–pituitary–adrenal (HPA) axis and autonomic activation, stress can induce pro-inflammatory responses in the brain and periphery including a complex release of cytokines and chemokines [2], prostanoids, free radicals and transcription factors [3]. Stress-related effects on the immune system are increasingly linked to adverse health risks [4,5] and to increased neuroinflammation [6,7], which, in turn, is implicated in neural pathogenesis [8,9], and neuropsychiatric disorders [10,11]. Stress-induced “priming” or sensitization of the neuroinflammatory response can increase vulnerability to subsequent pro-inflammatory challenges [12]. Stress and increased neuroinflammation can also impair executive functions [13] with probable impact on astronaut performance.

Astronauts on the planned Mars missions will receive predicted exposure to ~13 cGy/yr space radiation (SR) and will likely face psychological stressors including social isolation (SI). SR and SI can have deleterious effects on physical and mental health, and both have been reported to alter immune system function. SR exposure results in marked and persistent gliosis [14,15,16] and microglia activation [17,18]. SI alone induces neuroinflammation and microglial overactivation [19], increases redox stress and pro-inflammatory TNFα levels in the hippocampus (HPC) [20], and reduces PV+ interneurons in the CA2 and CA3 regions of HPC [21]. SR and SI also may interact in ways that are not fully understood and alter immune function in different ways than acting alone. Effects may also vary with individual differences in stress resilience and vulnerability.

The need to understand individual differences in the ability to cope with stress and the effects of mixed stressors is being widely recognized (e.g., [22]). Much of the focus has been on understanding adaptive and maladaptive stress responses and in the context of pathological changes in mental and physical health. Understanding these factors also will be of significant importance for astronauts who will experience stressors in a unique and harsh environment that can impact their health and mission-related performance. Individual differences in the impact of SR on performance has been found in studies in rats which showed considerable inter-individual susceptibility and resilience to SR induced impairments of executive function, with ~30% (ATSET performance) or 60% (rodent psychomotor vigilance test (rPVT)) of rats showing apparently normal performance and the remaining rats performing the tasks poorly [23,24,25]. SI also can negatively impact cognitive performance [26,27] though potential individual differences in effects have received minimal attention. Rats can also show significant individual differences in response to stressors. We have demonstrated that vulnerable (Vul) outbred Wistar strain rats show significant reductions in rapid eye movement sleep (REM) after shock training in a conditioned fear paradigm whereas resilient (Res) rats do not. Responses to fearful contexts are virtually identical [28,29,30]. The rapidly normalized and enhanced REM in Res rats suggests a more rapid restoration of sleep homeostasis [30], which is consistent with stress resilience. Res and Vul rats also differ in behavioral responses to SI and SR [31,32]. Thus, determining how SI and SR, and their interactions impact immune function is crucial for fully understanding the pathways by which inflight stressors impact astronaut health and performance. It will also be important to understand how these responses can vary across individuals.

Here, we used ground-based models in rats to determine how SI, SR, and dual flight stressors (DFS: SI + SR), modified immune cell recruitment into the brain and altered gene expression related to immune signaling and neuroinflammation, and their relevance to brain homeostasis. We also assessed whether the effects of SI, SR, and DFS differed in putative Res and Vul rats as determined by their sleep responses to stress [28,29,30].

## 2. Materials and Methods

### 2.1. Subjects

Male, outbred, Wistar strain rats (8–9 months old at start of study) obtained from Hilltop Lab Animals, Inc. (Scottdale, PA, USA) were used in this study. Some cohorts of rats received a single dose of SR (15 cGy simplified 5-ion galactic cosmic radiation (GCRsim), Brookhaven National Laboratory (BNL); Long Island, NY, USA). Some non-irradiated animals were shipped with the SR-treated groups to control for possible effects on sleep and behavior caused by the additional handling. We observed no significant differences in behavior or sleep between animals maintained in house and the Sham groups that were shipped to BNL (either Control or SI) [31]. The groups were subsequently combined for the final analyses.

Prior to assignment to experimental groups, all rats were individually housed in transparent cages. At assignment, rats were randomly selected and subjected to either SI (opaque barriers between cages) or continued to be individually housed in transparent cages (as a Control group). SI was implemented beginning 35 days after SR in irradiated rats (DFS group) and at an equivalent time in non-irradiated rats (SI group). Subsequently, the animals were undisturbed other than health checks and husbandry for at least eight weeks prior to beginning experiments. After SI began, all housing conditions were maintained throughout the study as previously described [31]. Food and water were continuously available. The animals were kept on a 12:12 light:dark cycle and ambient temperature was maintained at 24.5 °C ± 0.5 °C.

All experiments were conducted following the National Institutes of Health Guide for the Care and Use of Experimental Animals and were approved by Eastern Virginia Medical School’s Institutional Animal Care and Use Committee (Protocol#: 19-018).

### 2.2. Determination of Res and Vul Subgroups

Prior to euthanasia, the rats were implanted for recording sleep via telemetry and then trained in a conditioned fear paradigm with sleep recording after training and testing. The rats were then classified as either Res or Vul based on percent change in REM amounts following footshock training compared to baseline REM amounts [28,29,30]. Vul rats exhibited a 50% or greater decrease in REM during the first 4 h of post-shock recording compared to baseline. Res rats had ≤50% decreases, no change in REM, or increases in REM compared to baseline. These criteria determined the Res and Vul phenotypes used in this study (n = 4–5 Res or Vul rats per comparison). Res and Vul groups were determined in a blinded fashion and no animals were excluded from the analyses.

### 2.3. Euthanasia

Rats were euthanized via isoflurane sedation (inhalant: 5%, ≤5 min duration) and perfused through the heart with 1X PBS. Their brains were removed and separated along the longitudinal fissure. The left hemisphere was used for analyses provided in a separate contribution [33]. The right hemisphere was prepared as described in detail below.

### 2.4. RNA Extraction

For a subset of animals (n = 9 in each group), the right half of the brain was snap frozen and stored in RNAlater (ThermoFisher Scientific, Waltham, MA, USA) at −80 °C until RNA isolation occurred using the Qiagen RNeasy Mini Kit, Hilden, Germany (Cat. #74104).

### 2.5. Nanostring Assay

Total RNA samples from each half brain were loaded into Nanostring^®^ Mouse Neuroinflammation Panels, which contain a set of over 770 pre-selected mouse genes related to neuroinflammatory and immune processes (332 of these gene markers are cross-reactive with rat). Results from the panels were analyzed using the nSolver database (Version 4.0.70; Nanostring Technologies; Seattle, WA, USA) and gene and pathway profiles for the groups were compiled and assessed for differences in neuroinflammatory-related expression levels.

Genes in each assay were divided by an appropriate internal reference gene and averaged to generate normalized counts. Fold changes were determined relative to basal levels detected in Control-Res animals and relative fold changes in transcript levels for each determined gene were compared between groups. The data were analyzed within nSolver using multiple t-tests with a Benjamini-Yekutieli correction. Phenotypes (Res and Vul) within each Treatment (Control, SI, SR, and DFS) were evaluated for potential differences. Within nSolver, genes were tested for differential expression across treatments using a single linear regression fit for each individual gene to predict expression and then grouped by biological pathway. Pathway regulation scores were determined using the nSolver database via directed global significance scores of overlaid differential gene expression data for sets of genes grouped by biological function relative to the Control-Res animals. The analysis measured the extent to which genes within a given set were differentially regulated compared to the independent variable.

### 2.6. Cell Isolation

In another subset of animals (n = 3–4 in each group), the right half of the brain was minced and enzymatically digested using the Miltenyi^®^ (Auburn, CA, USA) Adult Brain Dissociation Kit for mouse and rat (Cat. #130-107-677). Briefly, the minced brain tissue was placed in the gentleMACS^®^ C Tubes (Gaithersburg, MD, USA) (Cat. #130-096-334) along with the kit components and placed on the Miltenyi^®^ gentleMACS^®^ Octo Dissociator with Heaters (Cat. #130-096-427) for mechanical dissociation during the enzyme incubation step. After dissociation, the debris removal solution (Cat. #130-109-398) was used to eliminate debris (e.g., lipids and myelin), and red blood cell lysis solution (Cat. #130-094-193) was used to remove erythrocytes. Isolated cells were then re-suspended in a 90% BSA 10% DMSO freezing media solution and stored at −80 °C until further analysis took place.

### 2.7. Flow Cytometry

Frozen cell isolates were thawed in a 37 °C water bath, then kept on ice. Total viable cell numbers were counted using 0.4% trypan blue (Gibco-ThermoFisher Scientific, Grand Island, NY, USA). Single cell suspensions were stained in 1X HBSS supplemented with 5% FBS. The following monoclonal antibodies were used at a 1:100 dilution factor unless otherwise indicated for spectral flow cytometric analysis: CD4 (Miltenyi, clone REA482, cat. #130-123-286), CD8a (Miltenyi, clone REA437, cat. #130-108-878), CD3 (Miltenyi, clone REA223, cat. #130-127-365, 1:400), CD11b/c (Miltenyi, clone REA325, cat. #130-105-273), CD45RA (Biolegend, San Diego, CA, USA, clone OX-33, cat. #202318), MHCII (Miltenyi, clone REA564, cat. #130-108-711), CD25 (Biolegend, clone OX-39, cat. #202105, 1:200 dilution), CD45RC (Miltenyi, clone REA1000, cat. #130-116-903), CD134 (Miltenyi, clone REA540, cat. #130-108-188, 1:50 dilution), CD71 (Biolegend, clone OX-26, cat. #204412), CD43 (Biolegend, clone W313, cat. #202810), CD45 (Biolegend, clone OX-1, cat. #202218, 1:200 dilution), and CD86 (Miltenyi, clone 24F, cat. #130-109-132, 1:200 dilution). Data were acquired on a Cytek^®^ Aurora 4L 16V-14B-10YG-8R spectral flow cytometer and analyzed with Cytek SpectroFlo^®^ (Cytek Biosciences; Fremont, CA, USA). Cells were identified with the following gating scheme: time gate, doublet/aggregate exclusion, and cells. Viable cells were identified as unstained with Zombie-NIR (Biolegend, cat. #423105). Cells were identified as (i) resident cells (CD11b-CD45-), (ii) infiltrating myeloid cells (CD11b+CD45+), and (iii) infiltrating lymphoid cells (CD11b-CD45+). Further analysis to delineate specific cell populations was hindered due to complications with the remaining antibodies and the lack of commercially available rat-specific antibodies, alternative clones were unavailable.

### 2.8. Statistical Analyses

Data that did not consider the Res/Vul phenotype separation were analyzed with a one-way ANOVA between Treatments (Control, SI, SR, and DFS). Post hoc comparisons were performed following significant ANOVAs using Tukey’s tests. All statistics were performed using GraphPad PRISM software (Version 9.4.1). Statistical assumptions were tested as a standard procedure in the analysis.

## 3. Results

### 3.1. Immune Cell Infiltration

**Figure 1:**
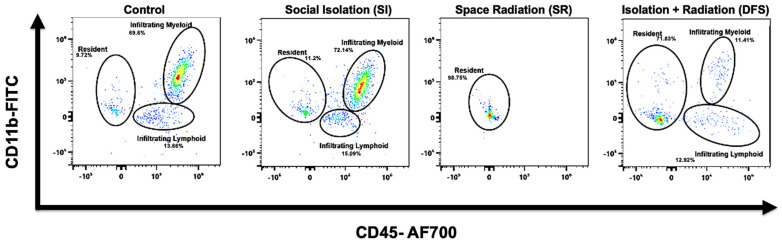
Irradiated animals had a loss of infiltrating immune cells to the brain. Pseudoplots displaying differences in resident and infiltrating cell populations in the brain in each treatment group. After live cell gating, resident cells were classified as CD11b-CD45-; infiltrating myeloid cells were classified as CD11b+CD45+; and infiltrating lymphoid cells were classified as CD11b-CD45+.

**Figure 2:**
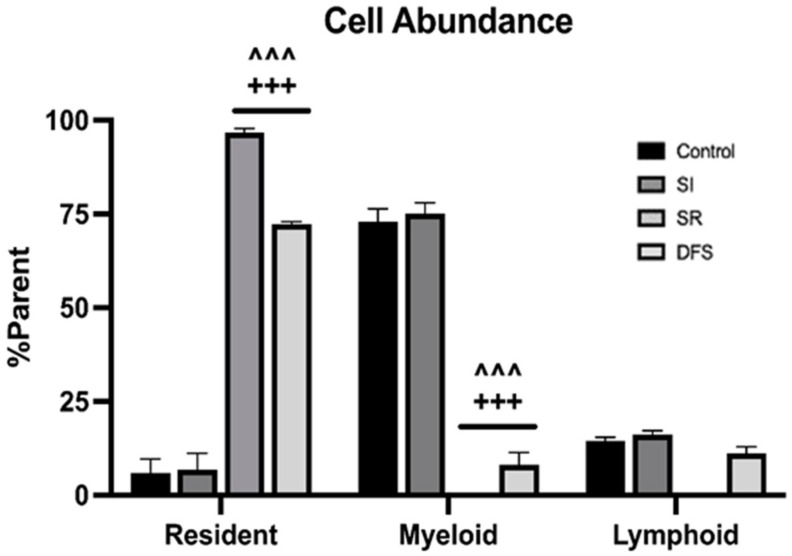
Resident and infiltrating cell populations differed between treatment groups. Graph plotting the relative % parent ± SEM of resident, myeloid, and lymphoid cell populations in each treatment group. +++ *p* < 0.001, compared to Control; ^^^ *p* < 0.001, compared to SI. SI: social isolation; SR: space radiation; DFS: SR + SI.

Flow cytometry revealed that Control and SI animals had high levels of infiltrating CD45+ myeloid and CD11b+CD45+ populations within the brain, but these were depleted in SR animals. Interestingly, we observed a slight rescue of these infiltrating cell populations in the DFS treatment animals consistent with other metrics we investigated (Figure 1). An additional interesting observation within the resident cell cluster was the presence of a CD11b+ population that was present in all groups except for the SR group. This population of cells could be resident microglia; however, due to the lack of appropriate markers, as discussed above, this is unconfirmed (Figure 1). ANOVA revealed significant differences for Treatment (F3,24 = 233.5; *p* < 0.0001). Tukey’s post hoc tests revealed that SR and DFS animals had significantly higher percentage of resident cells compared to the parent cell population than Control and SI groups (*p* < 0.001 for both comparisons; Figure 2). In contrast, Control and SI animals also had significantly higher percentage of infiltrating myeloid cells compared to the parent cell population than did the SR and DFS groups (*p* < 0.001 for both comparisons; Figure 2). While the Control, SI, and DFS treated animals each had an apparent increase in the infiltrating lymphoid cell population compared to the SR treated animals, the difference was not found to be significant for any comparisons.

### 3.2. Gene Expression Related to Neuroinflammatory Signaling

**Figure 3:**
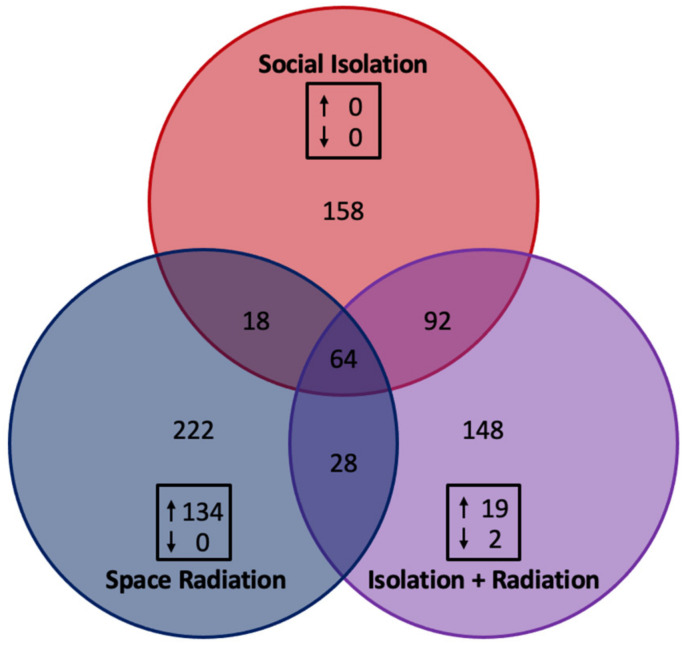
Gene expression was differentially modulated by social isolation and space radiation. Diagram displaying the number of unique versus shared genes between each treatment group irrespective of Res and Vul phenotype, and how many genes were significantly up- or down-regulated (within the black box) compared to Control.

Investigation into the number of differentially regulated genes revealed 158 genes in SI treated animals, 222 genes in SR treated animals and 148 genes in DFS treated animals that were uniquely regulated (Figure 3). Of the remaining genes, 18 genes were found to be similarly regulated between the SI and SR treated animals; 92 genes were found to be similarly regulated between the SI and DFS treated animals; 28 genes were found to be similarly regulated between the SR and DFS treated animals; and 64 genes were found to be similarly regulated between the three treatments (Figure 3). However, the built-in nSolver analysis found that, compared to the Control group, 0 genes were significantly different in SI treated animals, 134 genes were significantly different in SR treated animals (all up-regulated), and 21 genes were significantly different in DFS treated animals (19 up-regulated and 2 down-regulated) (Figure 3).

**Figure 4:**
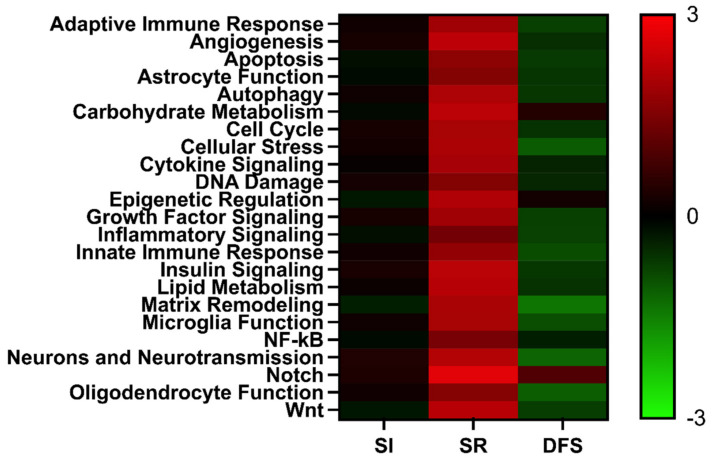
Inflight stressors differentially influenced inflammatory-related pathways in the brain. Heatmaps showing the directed global significance scores for sets of genes grouped by biological function relative to Control. Directed global significance examines the extent to which a set of genes is up- or down-regulated with a treatment. Red indicates gene sets that show over-expression and green indicates gene sets that show under-expression. SI: social isolation; SR: space radiation; DFS: SR + SI. SI and SR treated animals (*p* < 0.001 for all comparisons); SR and DFS treated animals s (*p* < 0.001 for all comparisons); SI and DFS treated animals (no significant differences).

Nanostring^®^ Mouse Neuroinflammatory Panels revealed that SI, SR, and DFS differentially regulated pathways related to neuroinflammation based on gene expression markers relative to Control. ANOVA revealed significant differences between Treatment (F2,44 = 362.0; *p* < 0.0001) and significant differences between pathways (F22,44 = 1.790; *p* = 0.04). Tukey’s post hoc revealed significant differences between SI and SR treated animals (*p* < 0.001 for all comparisons) and significant differences between SR and DFS treated animals (*p* < 0.001 for all comparisons). No significant differences were found between SI and DFS treated animals (Figure 4).

### 3.3. Gene Expression in Res Versus Vul Animals

**Figure 5:**
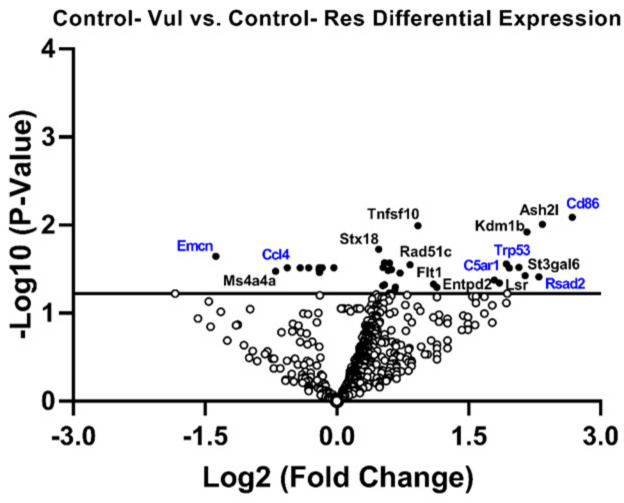
Control vulnerable (Vul) animals showed increased expression of inflammatory- and immune response-related genes. Volcano plot displaying gene expression levels in the brains of Vul animals compared to Control resilient (Res) animals. Genes above the horizontal line are significant and those to either side of the zero on the *x*-axis are differentially expressed. The most relevant genes are labeled and genes discussed in the text are indicated in blue.

Examining the specific expression of each gene compared to Control-Res animals revealed that Control-Vul animals showed an up-regulation of genes related to inflammatory signaling (e.g., C5ar1, *p* = 0.02; Rsad2, *p* = 0.05) and the adaptive immune response (e.g., Cd86, *p* = 0.05; and), and apoptosis (e.g., Trp53, *p* = 0.02). Moreover, Control-Vul animals showed a down-regulation of genes related to matrix remodeling (e.g., Emcn, *p* = 0.01), and the innate immune response (e.g., Ccl4, *p* = 0.05) (Figure 5).

**Figure 6:**
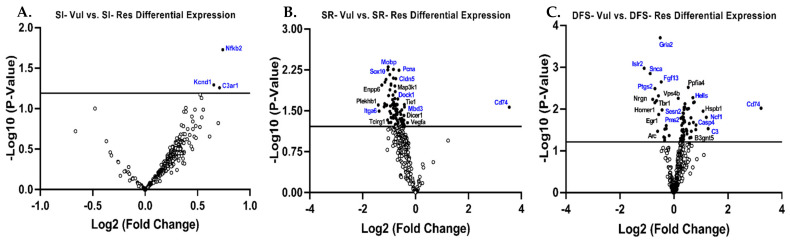
Gene expression within treatment groups differed between phenotypes. Volcano plot displaying gene expression levels in the brain of (**A**) Social isolation (SI) vulnerable (Vul), and (**B**) Space radiation (SR) Vul, and (**C**) Dual flight stressor (DFS) Vul animals compared to their resilient (Res) counterparts. Genes above the horizontal line are significant and those to either side of the zero on the *x*-axis are differentially expressed. The most relevant genes are labeled and genes discussed in the text indicated in blue.

Changes in gene expression in Vul animals compared to Res animals were also assessed within each treatment group (SI, SR, and DFS). Minimal differences were found between SI Vul and Res animals apart from three genes, one related to inflammatory signaling (e.g., Nfkb2, *p* = 0.02) and two genes related to immune cell function (e.g., Kcnd1, *p* = 0.05; and C3ar1, *p* = 0.05) (Figure 6A). However, gene expression between SR and DFS Vul and Res animals was starkly different. Compared to SR Res animals, SR Vul animals exhibited increased expression of one gene related to inflammatory signaling (e.g., Cd74, *p* = 0.03). SR Vul animals exhibited decreased expression of genes related to neurotransmission (e.g., Sox10, *p* < 0.01 and Mobp, *p* < 0.01), angiogenesis (e.g., Dock1, *p* = 0.01), epigenetic regulation (e.g., Mbd3, *p* = 0.02), matrix remodeling (e.g., Cldn5, *p* = 0.03), growth factor signaling (e.g., Itga6, *p* = 0.02), and cell cycle regulation (e.g., Pcna, *p* < 0.01) (Figure 6B). Compared to DFS Res animals, DFS Vul animals exhibited an up-regulation of genes related to inflammatory signaling (e.g., Cd74, *p* < 0.01), the adaptive immune response (e.g., Ncf1, *p* = 0.01 and C3, *p* = 0.03), DNA damage (e.g., Pms2, *p* < 0.01 and Sesn2, *p* = 0.01), and apoptosis (e.g., Hells, *p* < 0.01 and Casp4, *p* = 0.02). DFS Vul animals further exhibited a down-regulation of genes related to neurotransmission (e.g., Gria2, *p* = 0.0001 and Islr2, *p* = 0.001), astrocyte function (e.g., Ptgs2, *p* = 0.003), microglia function (e.g., Snca, *p* = 0.001 and Fgf13, *p* < 0.01) (Figure 6C).

### 3.4. Alterations in Brain Neuroimmune Pathways

**Figure 7:**
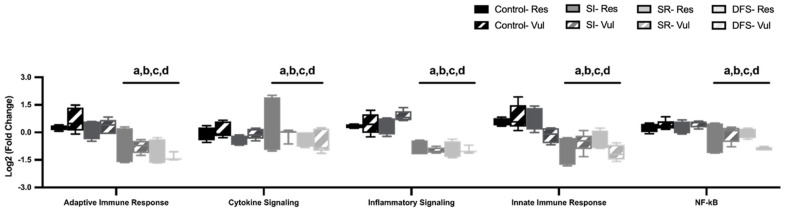
Irradiated animals had decreased neurotransmission and depressed immune-related pathways. Graphs displaying pathways affiliated with neuroimmune response (adaptive immune response, cytokine signaling, inflammatory signaling, innate immune response, and NFkB) for resilient (Res) and vulnerable (Vul) animals in each treatment group. Significant differences (*p* < 0.05) compared to: a, Control-Res; b, Control-Vul; c, SI-Res; d, SI-Vul.

Despite the up-regulation of individual genes, overall pathways related to the neuroimmune response (adaptive immune response, cytokine signaling, inflammatory signaling, innate immune response, and NFkB) were found to be significantly downregulated. ANOVA revealed significant differences between Phenotype (F7,160 = 17.29; *p* < 0.0001). Tukey’s post hoc revealed that SR and DFS animals had significant decreases in multiple pathways related to the neuroimmune response (*p* < 0.05 compared to Control and SI groups for all pathways) (Figure 7).

## 4. Discussion

In the current study, we found a suppressed immune response in the SR treatment group. This was associated with a depletion of infiltrating immune cells, despite up-regulated gene expression related to cytokine signaling and immune cell recruitment. Interestingly, many deficits related to the immune response observed in the SR treated animals were attenuated by dual exposure to SI (DFS). The treatment group that was exposed to SI alone exhibited differences in neuroinflammatory signaling and BBB integrity (reported previously [33]). However, differences in neuroinflammatory signaling were not associated with differences in immune infiltration compared to Control. Nanostring^®^ panels showed increased pro-inflammatory signaling in the SI, SR, and DFS treatment groups, but only the SR and DFS treatment groups showed an increased expression of repair mechanisms. Many of these repair mechanisms were related to vascular regulation, matrix remodeling, and growth factor signaling, which were likely activated due to the increased BBB damage seen in the SR and DFS treatment groups [33].

SR animals had a virtually complete loss of peripheral infiltrating cells in the brain compared to Control and SI animals. This finding could possibly help explain the decrease in immune-related pathways observed within the irradiated treatment groups (SR and DFS). Previous reports have shown altered immune system functionality in astronauts after international space station (ISS) missions and studies have found that aspects of both the innate and adaptive immune system are impaired during spaceflight [34,35]. However, these studies focused on measurable outcomes from astronauts on the ISS that were exposed to a myriad of spaceflight stressors rather than elucidating specific causations. Our study may be the first to show a complete loss of immune cell viability by SR alone. This finding was somewhat unexpected as Nanostring^®^ panels showed that SR animals had a major up-regulation of many genes related to pro-inflammatory signaling and immune cell recruitment. One plausible explanation for this increase in gene expression may be that the system’s feedback loop was disrupted (due to cell death). Without a feedback response from these immune cells, the only action the system could perform was to increase the intensity of the signals being released. If so, we could also expect a depletion of bone marrow which is the most radiation sensitive organ in the body [36] and other groups are currently investigating how SR exposure can alter the microstructure and the contents of bone [37,38,39]. However, two alternative scenarios, rather than complete cell death/bone marrow depletion, could also explain the lack of immune infiltrate in SR animals. One, SR could negatively affect the motility of these immune cells and prevent them from migrating the long distances to the brain. Two, there may be a disruption of protein synthesis that compromises or prevents proper signaling. Both scenarios could also account for the up-regulation of the gene markers seen within Nanostring^®^. Future studies will be needed to investigate these mechanisms to confirm or refute these hypotheses.

Interestingly, DFS animals exhibited a slight rescue of peripheral immune cells that migrated to the brain, but amounts were still significantly less compared to Control and SI animals. To our knowledge, this study is the first demonstration that dual exposure of SI with SR ameliorated immune cell loss induced by SR alone. The synergistic relationship between SI and SR on the immune system is currently unknown. However, SI alone induces neuroinflammation and microglial overactivation [19], and it increases redox stress and pro-inflammatory cytokines [20]. Our study demonstrated that SI animals had higher levels of pro-inflammatory signaling markers in the Nanostring^®^ panels and slightly higher levels of immune infiltrate in the brain compared to Control animals. Therefore, it is possible that the chronic SI may have induced a low-grade inflammatory environment which primed the immune system and rescued some cells from SR-induced damage or death. It is also possible that SI promoted an adaptive response, which was protective from the functional deficits produced by SR [40]. However, these hypotheses will require further testing and will be another direction for future studies.

It also is essential to point out that SI was implemented five weeks after SR in the current study. The order that astronauts experience spaceflight stressors may differ within the time course of their mission which, in turn, may impact synergistic interactions among stressors and their effects on mission-related performance. The order, duration, and ability to resolve a given stressor are important as prolonged stress and the failure to appropriately resolve a physiological stress response can increase vulnerability to subsequent stress [41]. Thus, it will be vital to also test the effects of SR administered concurrently with SI as the effects of SR may vary depending on the physiological status of astronauts at the time of irradiation. SI can reduce the ability to withstand an immune system challenge [42,43] and reduce the amount and efficiency of sleep [44,45,46], both of which could impact the ability to withstand SR. SI can also increase the risk for myocardial infarctions [47], general morbidity and mortality [48,49], and have other negative effects on general human health which could impact astronaut performance.

Stress resilience will play a key role in deep space missions. Astronauts are highly selected for physical and mental performance and also are highly trained which may increase resilience to some aspects of spaceflight stress. Indeed, resilience can involve behaviors, thoughts, and actions that can be learned and developed [50]. Social support is also important for an individual’s ability to cope with stress [51], whereas a lack of social support may increase the negative impact of stress. Previous studies have characterized Vul individuals as being more susceptible to immune changes during stress [41,52]. In general, our results substantiate the need to maintain strong social support on space missions and suggest that the physical effects of SR may dramatically alter even Res individuals abilities to cope with stress, particularly the prolonged and unrelenting stressors that astronauts will likely face. The somewhat unexpected “protective” effect found in DFS rats will need further research to understand though as indicated it may involve chronic low-grade inflammation or an adaptive response not found in Control animals.

This work has limitations that should be addressed. First, while we observed differences in peripheral immune cell recruitment and resident cells within the brain, the lack of quality rat-reactive antibodies for flow cytometry experiments greatly limited our ability to further characterize specific populations and their involvement. This will be of great relevance for future experiments to determine specific immune cell interactions in response to SI and SR. While Nanostring^®^ panels revealed many differences between treatment groups, they are specifically designed for mouse mRNA samples. As such, only 332 genes in the panel were cross-reactive with rat genes. This limited the amount of information that could assess specific pathways that could lead to changes in morphology and BBB permeability that we also observed [33]. Additionally, it is possible that current data obtained from Nanostring^®^ was skewed based on differences in cross-reactivity. Therefore, results from Nanostring^®^ will need validation by alternative techniques (i.e., ELISA, PCR, Southern Blot, etc.).

It should also be mentioned that while using differences in REM sleep as an index of resilience and vulnerability is based on both conceptual and behavioral outcomes [53], these differences may not fully reflect differences in stress resilience. Differences in resilience and vulnerability may also involve differences in immune system responses [53] which we saw in this study. However, the small sample sizes (n = 4–5 per group) as well as the lack of rat specific antibodies indicate that the current data should not be considered unequivocal. Future studies with greater numbers of subjects and rat specific antibodies, or similar studies in mice, will be required to more fully delineate the potential effects of SR and other spaceflight stressors in Vul and Res animals. However, the current data suggest that SR can produce differential immune responses in Vul and Res rats that also show differences in sleep and behavioral measures [31,32].

## 5. Conclusions

SI [19,20,21], SR [14,15,16,17,18], and their interactions can alter immune function, which may further exacerbate the effects of stress. Effects may also vary with individual differences in stress resilience and vulnerability. In this study, we show that both SI and SR differentially altered gene expression related to immune functionality and neuroinflammation, and that SR caused a dramatic loss of immune cell infiltrate. This loss was slightly rescued by dual exposure of SI with SR (DFS), indicating SI and SR interactions can change immune system responses, and potentially provide a model for delineating pathways that could protect against the effects of SR. Additionally, compared to Res animals, Vul rats are more likely to develop physical and cognitive deficits and show different immune responses to SI and SR; thus, indicating that individual differences should be considered in assessing their effects on astronaut health and performance.

## Data Availability

Experimental data available upon request.

## Abbreviations

The following abbreviations are used in this manuscript:

DFS	dual flight stressors
HPA	Hypothalamic–Pituitary–Adrenal
SR	space radiation
SI	social isolation
HPC	hippocampus
ATSET	Attentional Set-Shifting
rPVT	rodent psychomotor vigilance test
Vul	vulnerable
REM	rapid eye movement sleep
Res	resilient
GCRsim	15 cGy simplified 5-ion galactic cosmic radiation
ISS	international space station
